# Induction of Interleukin-10 Producing Dendritic Cells As a Tool to Suppress Allergen-Specific T Helper 2 Responses

**DOI:** 10.3389/fimmu.2018.00455

**Published:** 2018-03-19

**Authors:** Stefan Schülke

**Affiliations:** ^1^Vice President’s Research Group 1, Molecular Allergology, Paul-Ehrlich-Institut, Langen, Germany

**Keywords:** dendritic cells, interleukin-10, allergy, T helper 2, dendritic cell vaccine

## Abstract

Dendritic cells (DCs) are gatekeepers of the immune system that control induction and polarization of primary, antigen-specific immune responses. Depending on their maturation/activation status, the molecules expressed on their surface, and the cytokines produced DCs have been shown to either elicit immune responses through activation of effector T cells or induce tolerance through induction of either T cell anergy, regulatory T cells, or production of regulatory cytokines. Among the cytokines produced by tolerogenic DCs, interleukin 10 (IL-10) is a key regulatory cytokine limiting und ultimately terminating excessive T-cell responses to microbial pathogens to prevent chronic inflammation and tissue damage. Because of their important role in preventing autoimmune diseases, transplant rejection, allergic reactions, or in controlling chronic inflammation DCs have become an interesting tool to modulate antigen-specific immune responses. For the treatment of allergic inflammation, the aim is to downregulate allergen-specific T helper 2 (Th2) responses and the associated clinical symptoms [allergen-driven Th2 activation, Th2-driven immunoglobulin E (IgE) production, IgE-mediated mast cell and basophil activation, allergic inflammation]. Here, combining the presentation of allergens by DCs with a pro-tolerogenic, IL-10-producing phenotype is of special interest to modulate allergen-specific immune responses in the treatment of allergic diseases. This review discusses the reported strategies to induce DC-derived IL-10 secretion for the suppression of allergen-specific Th2-responses with a focus on IL-10 treatment, IL-10 transduction, and the usage of both whole bacteria and bacteria-derived components. Interestingly, while IL-10-producing DCs induced either by IL-10 treatment or IL-10 transduction are arrested in an immature/semi-mature state, treatment of DCs with live or killed bacteria as well as isolated bacterial components results in the induction of both anti-inflammatory IL-10 and pro-inflammatory, Th1-promoting IL-12 secretion often paralleled by an enhanced expression of co-stimulatory molecules on the stimulated DCs. By the secretion of DC-derived exosomes or CC-chemokine ligand 18, as well as the expression of inhibitory molecules like cytotoxic T lymphocyte-associated antigen 4, TNF receptor superfamily member 4, Ig-like transcript-22/cluster of differentiation 85, or programmed death-1, IL-10-producing DCs have been repeatedly shown to suppress antigen-specific Th2-responses. Therefore, DC-based vaccination approaches hold great potential to improve the treatment of allergic diseases.

## Introduction

### Dendritic Cells (DCs) Control the Induction of Immune Responses

Our immune system efficiently protects us from most pathogens. However, if the actions of immune cells are misdirected (e.g., against our own cells and tissues in the case of autoimmune diseases or against innocuous environmental antigens in the case of allergies) severe immunopathology can be the consequence. Therefore, the induction of potentially highly destructive immune responses needs to be tightly regulated.

Usually, the prevention of such detrimental immune responses is achieved by controlling against which antigens cells of the adaptive immune system are allowed to react. Here, antigen-presenting cells (APCs) are pivotal in controlling the induction of innate and subsequent adaptive immune responses.

Antigen-presenting cells consist of DCs, macrophages, and B cells (1, 2). They control both the induction and regulation of T-cell immune responses *via* the uptake, processing, and presentation of antigens to antigen-specific T cells (1, 2).

Among the different types of APCs, DCs are of special importance because they are the only APC type able to induce activation, differentiation, and expansion of naive, antigen-specific T cells (3, 4). In contrast to this, macrophages and B cells are only sufficient to reactivate T cells that have already encountered their specific antigen in the past (5).

Dendritic cells are highly specialized APCs strategically located in the skin and the mucosal system (2, 6). They act as sentinel cells that initiate, monitor, and regulate immune responses (1). In their immature form DCs continuously take up and process antigens *via* endocytosis or pinocytosis (7). If this antigen uptake occurs in the context of additional DC-activating signals such as pro-inflamatory cytokines [tumor necrosis factor alpha (TNF-α), interleukin (IL)-1β, or IL-6], prostaglandin hormones (prostaglandin E 2), immune stimulating bacterial and viral components [lipopolysaccharide (LPS), CpG-DNA; Pam_2_CysK_4_, flagellin, etc.], or cell-contact-dependent signals [e.g., *via* cluster of differentiation (CD)40-ligand] DCs become activated (8). Once activated, DCs start to present the processed antigens in the context of major histocompatibility complex II (MHC II) molecules and express co-stimulatory molecules on their surface (2, 8). *Via* the expression of the chemokine receptor 7 (CCR7, whose ligand is abundantly expressed in lymph nodes) mature DCs also start to migrate to lymph nodes, where DCs encounter antigen-specific naive T cells and initiate their priming (9, 10).

By their actions, DCs link innate and adaptive immune responses by connecting the detection of danger signals with the uptake, processing, and presentation of foreign antigens to control both the induction and polarization of primary antigen-specific CD4^+^ T-cell responses (11, 12).

Besides their important function in the induction of antigen-specific immune responses, DCs are also key players in maintaining immune homeostasis (13). Uptake and presentation of innocuous foreign- and self-antigens by DCs usually mediates T-cell tolerance (14). In this context, the cytokine IL-10 has been shown to shift DC function toward a tolerogenic rather than an immunogenic phenotype (15).

Dendritic cells may acquire tolerogenic properties either by (1) displaying a semi-mature state and exert tolerogenic function *via* the induction of apoptosis or anergy in the absence of co-stimulatory signals (2, 3, 16) promoting the differentiation of interacting T cells into CD4^+^CD25^+^ regulatory T (Treg) cells, or (3) increasing IL-10 production to expand allergen-specific type 1 regulatory T (Tr1) cells (3, 17, 18). Indeed, the T cell skewing capacity of DCs largely depends on their cytokine pattern and expression of co-stimulatory molecules (19, 20).

Therefore, depending on their maturation/activation status, the molecules expressed on their surface, and their cytokine production DCs have been shown to elicit immune responses through either activation of effector T cells, induction of tolerance through regulatory T cells, or the induction of regulatory cytokines (6).

Because of their important role in the induction of both innate and adaptive immune responses, DCs have become an interesting tool to modulate antigen-specific immune responses (11, 21). In this context, their capacity to induce, modulate, and control T cell responses makes DCs an attractive adjuvant in vaccination settings that have the aim to either enhance inadequate immune responses for the treatment of infectious diseases and cancer or to attenuate exaggerated immune responses in conditions such as autoimmunity, allergy, transplant rejection, and chronic inflammation (11, 21).

### IL-10 Is an Important Cytokine Limiting Excessive Immune Responses

As we have just seen, cytokines produced by DCs play a central role in controlling both the induction and polarization of primary antigen-specific T-cell responses.

Among the cytokines produced by DCs, IL-10 is a key regulatory cytokine limiting and ultimately terminating excessive T-cell responses to microbial pathogens to prevent chronic inflammation and tissue damage (15, 22). IL-10 can both be produced by and has pleiotropic effects on multiple cell types, including DCs, macrophages, B cells, natural killer cells, both Th1- and Th2 cells, CD4^+^CD25^+^ forkhead box protein 3 (Foxp3^+^) Treg cells, and keratinocytes (1, 23–25).

Interleukin-10, originally identified as an inhibitor of interferon gamma (IFN-γ) and IL-2 synthesis in Th2 cells (26), efficiently inhibits proliferative and cytokine responses in T cells (1) and was shown to mediate both immunological unresponsiveness and the suppression of immune reactions (27). At epithelial interfaces to the environment, including the skin, IL-10 prevents excessive immune responses to foreign antigens (25).

Indeed, a well-documented mechanism by which IL-10-producing DCs suppress allergic Th2-responses is the induction of allergen-specific CD4^+^CD25^+^Foxp3^+^ Treg cells (27–29). For example, Pacciani et al. reported that IL-10-producing DC can induce allergen-specific regulatory T cells suppressing proliferation and inflammatory cytokine production from Th2 cells of the same specificity from house dust mite-allergic patients (30). In line with this, Oh and colleagues showed that IL-10-secreting T cells in the airways were able to reduce Th2-type inflammation and airway hyperreactivity (AHR) (31). Therefore, the induction of IL-10- and transforming growth factor beta (TGF-β)-producing regulatory T cells by IL-10-producing DCs is an important mechanism to prevent excessive immune responses (32, 33).

Consequently, IL-10-deficient mice develop increased contact hypersensitivity (34), display spontaneous enterocolitis and other symptoms akin to Crohn’s disease (35), and develop exaggerated asthmatic and allergic responses (35).

**Figure 1:**
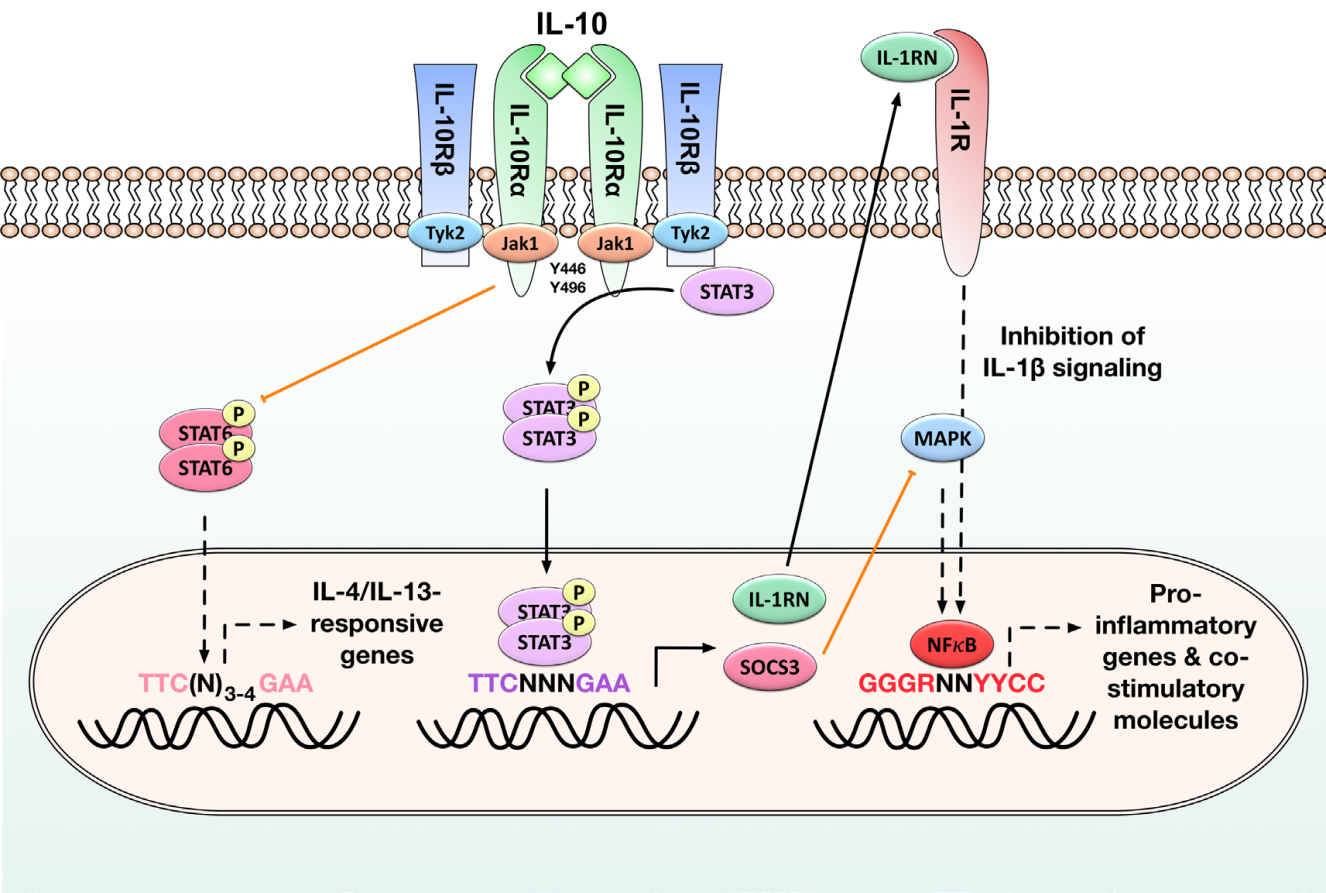
Immune modulatory signaling in antigen-presenting cells (APCs) induced by binding of interleukin-10 (IL-10) to the IL-10 receptor. Activation of the IL-10/Janus kinase 1 (JAK1)/tyrosine kinase 2 (Tyk2)/signal transducer and activator of transcription 3 (STAT3) pathway in APCs results in the phosphorylation of STAT3 by the interleukin 10 receptor (IL-10R) complex and the subsequent translocation of STAT3 homodimers into the nucleus. There STAT3 homodimers bind to STAT-binding elements and drive the expression of STAT-3-responsive genes such as suppressor of cytokine signaling 3 (SOCS-3) and IL-1 receptor antagonist (IL-1RN): SOCS-3 efficiently inhibits mitogen-activated protein kinase (MAP kinase) activation, NFκB translocation into the nucleus, and the subsequent induction of pro-inflammatory gene expression, while the decoy protein IL-1RN suppresses pro-inflammatory signaling normally initiated by binding of IL-1β to its receptor. STAT3 activation also inhibits STAT6 activation and therefore the expression of IL-4/IL-13-responsive genes. By these events, IL-10 reduces the production of pro-inflammatory cytokines (IL-1β, IL-6, tumor necrosis factor alpha) and diminished expression of both major histocompatibility complex II and co-stimulatory molecules (CD80, CD83, CD86) on APCs. Black arrows: activation of the indicated signaling pathways, orange arrows: inhibition of the indicated signaling pathways, black dashed arrows: pathways normally induced by the indicated molecules that are inhibited in the presence of STAT3 activation. For more detailed information, see Section “IL-10 Is an Important Cytokine Limiting Excessive Immune Responses.”

Interleukin-10 signaling is transmitted through a heterotetrameric interleukin 10 receptor (IL-10R) which consists of two ligand-binding IL-10R alpha chains and two accessory, signal-transducing beta chains all belonging to the interferon receptor family (Figure 1) (23). The IL-10R α chain is expressed at high levels on both macrophages and DCs, whereas the IL-10R β chain is ubiquitously and constitutively expressed by all cell types (23).

Mechanistically, IL-10 inhibits the function of APCs, including macrophages and DCs, by downregulating their maturation status and reducing the associated production of pro-inflammatory cytokines (such as IL-1β, IL-6, or TNF-α), while increasing the expression of inhibitory genes (23, 36). These effects of IL-10 are mediated *via* the Janus kinase 1 (JAK1)/Tyk2/STAT3 pathway. It is initiated when IL-10 homodimers bind to the extracellular portion of the IL-10R alpha chain (Figure 1). In a first activation step, IL-10 binding triggers the recruitment of Jak1 to the IL-10R alpha chain and its subsequent phosphorylation, while tyrosine kinase 2 (Tyk2) is recruited to and phosphorylated by the IL-10R beta chain (Figure 1) (37, 38). Upon their phosphorylation, these kinases phosphorylate the tyrosine motifs Y446 and Y496 located in the intracellular portion of the IL-10R alpha chain (Figure 1) (39). The activated IL-10 receptor complex then mediates the phosphorylation of signal transducer and activator of transcription 3 (STAT3) by providing transient anchorage sites for STAT-3 that allow the phosphorylation of STAT3 by Jak1 and Tyk2 (39, 40). Phosphorylated STAT3 forms homodimers which translocate into the nucleus, bind to STAT-binding elements, and drive the expression of STAT-3-responsive genes. Among others, these include the suppressor of cytokine signaling 3 (SOCS-3) and the IL-1 receptor antagonist (IL-1RN) (Figure 1) (41): SOCS-3 subsequently inhibits mitogen-activated protein kinase activation, NFκB translocation into the nucleus, and the associated induction of pro-inflammatory gene expression (40). SOCS-3 also mediates Jak1-inhibition, resulting in feedback inhibition of the JAK1/Tyk2/STAT3 pathway (42).

In addition, production of IL-1RN, a decoy protein binding to the IL-1 receptor, blocks pro-inflammatory signaling normally initiated by binding of IL-1β to this receptor (Figure 1) (43).

Interleukin-10 also directly inhibits Th1 cell differentiation (by reducing IL-2, IL-12, and INF-γ production), limits effector T-cell function (by suppressing TNF-α, IL-1β, and IL-6 production), and promotes the development, expansion, and function of regulatory T cells (23, 44). For example, IL-10 is known to inhibit the expression of IL-4 and IL-13-responsive genes in monocytes and DCs by suppressing the activation of STAT6 (Figure 1) (45).

Interleukin-10/IL-10R signaling may also result in STAT1 and STAT5 phosphorylation in monocytes and Treg cells, but the interactions of STAT1 and STAT5 with other intracellular signaling events triggered by IL-10 are still unclear (42).

Because of its broadly anti-inflammatory effects, IL-10 is a highly interesting molecule for the treatment of allergic diseases, where affected patients mount exaggerated, immunoglobulin E (IgE)- and Th2-mediated immune responses against otherwise harmless environmental antigens.

In line with this, DC-derived autocrine IL-10 secretion was shown to suppress high-affinity IgE receptor Fc epsilon receptor I-dependent pro-inflammatory responses (46), suggesting that increased IL-10 production by DCs during allergy immunotherapy may reduce inflammatory responses to the allergen (47).

Up to now, numerous studies support the importance of IL-10 produced by either Treg or Tr1 cells (48, 49), IL-10-producing regulatory B cells (50), and lung DCs (4, 32, 51) in the modulation of allergic diseases. Among other findings, IL-10 production by murine lung DCs suppressed inflammation and promoted the establishment of allergen-specific tolerance (52). In line with its function in the suppression of lung inflammation, IL-10 expression has been reported in DCs located in both lung tissue and the intestine, suggesting IL-10 to fulfill an important role in maintaining local T-cell tolerance to common environmental antigens (32).

The importance of IL-10 in controlling allergic inflammation is further highlighted by its ability to decrease eosinophil survival and IgE synthesis (53, 54). Indeed, IL-10 is often regarded as a key cytokine mediating tolerance in patients undergoing immunotherapy (55, 56).

### Allergic Patients Show a Tendency to Produce Reduced IL-10 Levels upon Allergen Contact

In line with the importance of IL-10 in suppressing allergic responses, Akbari and colleagues reported that DCs from mice exposed to harmless inhaled antigens transiently produce IL-10 stimulating the development of IL-10-secreting, antigen-specific CD4^+^CD25^+^Foxp3^+^ Tregs (32). Moreover, upon stimulation with the probiotic bacterium *Escherichia coli* 083, a lower expression and secretion of IL-10 was detected from monocyte-derived DCs (moDCs) derived from newborns of allergic mothers compared with cells derived from children with non-allergic mothers (57).

These results suggest that a reduced capacity to produce DC-derived IL-10 upon antigen contact may facilitate the development of allergic diseases by skewing immune responses toward the differentiation of Th2 cells and the development of childhood atopy and/or asthma (2).

Early studies showed that DC-derived IL-10 production is profoundly diminished in allergic rhinitis (AR) children regardless of the presence or absence of asthma, while DC-derived IL-12 secretion as well as T cell cytokine secretion were unchanged (2). These results suggest that atopic individuals have an intrinsic inability to upregulate DC-derived IL-10 production (2). In line with this, several studies reported diminished antigen-induced, peripheral blood mononuclear cell (PBMC)-derived IL-10 production in children (58, 59) and adults (31, 60, 61) with atopic disorders (AR, asthma, or atopic dermatitis).

In addition, several studies have reported that allergic patients show a tendency to produce reduced levels of IL-10 upon allergen exposure. For example, defects in IL-10-producing T cells have been implicated in the immunopathogenesis of airway allergy, resulting in Th2-mediated production of allergen-specific IgE and tissue eosinophilia (55, 62).

Moreover, Wei et al. reported that IL-10 levels in the supernatants of DCs from AR patients were significantly lower than those observed in healthy controls (63). Accordingly, Pilette and coworkers described not only local nasal DCs but also systemically circulating blood myeloid DCs (mDCs) from AR patients to exhibit reduced IL-10 and IL-12 expression after allergen provocation, while activated plasmacytoid DCs from these patients produced diminished amounts of interferon alpha (IFN-α) and triggered reduced levels of IL-10 from allogeneic CD4^+^ T cells (64). Due to these changes in cytokine production mDCs from AR patients preferentially supported Th2-cell polarization, linking systemic DC dysfunction to biased T-cell responses and the failure to regulate T-cell-mediated responses to allergens seen in atopic patients (64).

While most studies suggest allergic patients to produce reduced levels of IL-10 compared with healthy individuals, some groups report contrary results: Lied and coworkers reported LPS-stimulated moDCs from atopic patients to produce significantly more IL-10 compared to non-atopic patients (65) and Frischmeyer-Guerrerio and colleagues described that mDCs from food allergic children produced greater quantities of IL-10 (66).

### Successful Allergen-Specific Immunotherapy May Restore Reduced IL-10 Secretion in Allergic Patients

Since several studies have reported that allergic patients show a tendency to produce reduced levels of IL-10 upon allergen exposure (see above paragraph), restoring allergen-induced IL-10 secretion from DCs is one of the aims in allergen-specific immunotherapy (AIT).

Indeed, IL-10 is often regarded as a key cytokine mediating tolerance in patients undergoing immunotherapy (55, 56). Many of the observed beneficial immune alterations during AIT have been attributed to IL-10 production (67). Several studies reported increased levels of IL-10 in blood and affected tissues of patients that underwent AIT (4, 68, 69). However, the cellular source of AIT-induced IL-10 production seems to depend on the exact treatment modalities: for example, exposure to high doses of allergen was repeatedly shown to result in the induction of different IL-10-producing CD4^+^CD25^+^Foxp3^+^ Treg subsets (70–72), while for patients receiving pollen AIT increased IL-10 production was reported in mucosal macrophages (69). Interestingly, in contrast to this, increased numbers of IL-10-producing B cells and monocytes were described in the peripheral blood of patients receiving bee venom AIT (70, 73).

While many of the beneficial effects of AIT-induced IL-10 production are attributed to the induction of allergen-specific, IL-10-producing CD4^+^CD25^+^Foxp3^+^ Tregs (69–72), other cellular sources of IL-10 should not be ignored. In line with this, Kunz and coworkers recently described that the IL-10-dependent induction of allergen-specific tolerance by subcutaneous allergen injection resulted in increased IL-10 signals in T and B cells of both skin draining and mediastinal lymph nodes (74). Interestingly, tolerance induction could still be achieved when mice were unable to produce either T cell-, B cell-, T and B cell-, or DC-derived IL-10 (74). In contrast to this, tolerance induction was not possible if all hematopoetic cells were unable to produce IL-10 (74). Taken together, these results suggest a high degree of functional cellular redundancy in IL-10-mediated tolerance induction (74).

## Strategies to Induce DC-Derived IL-10 Secretion

**Figure 2:**
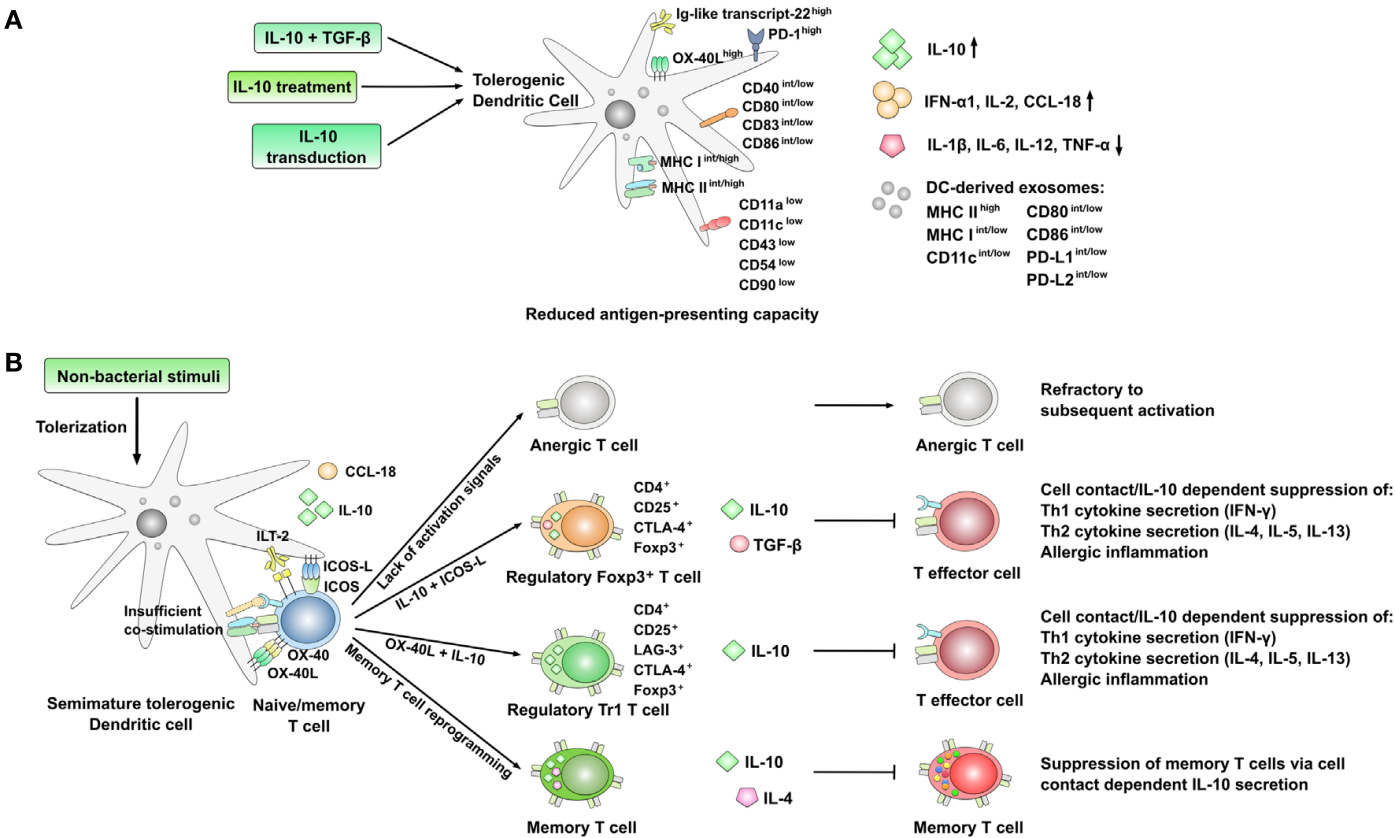
Phenotype and immune modulatory effects of interleukin-10 (IL-10)-producing, semi-mature tolerogenic DCs. **(A)** Strategies resulting in the generation of IL-10-producing, semi-mature tolerogenic dendritic cells (DCs) with reduced expression of co-stimulatory molecules, cell adhesion molecules, and lower secretion of pro-inflammatory cytokines. Expression levels are indicated as follows: ^low^: low expression, ^int^: intermediate expression, ^high^: high expression, ^+^: positive for the indicated molecule; arrow up: increased production, arrow down: decreased production. **(B)** Immune modulatory effects of tolerogenic DCs displaying a semi-mature DC phenotype. The lack of co-stimulation and antigen presentation results in the preferred induction of either anergic or regulatory T cell subsets which themselves are able to suppress both Th1- and Th2-responses. Also, the reprogramming of CD4^+^ memory T cells into IL-10 and IL-4 co-producing Th0-like cells has been described.

**Figure 3:**
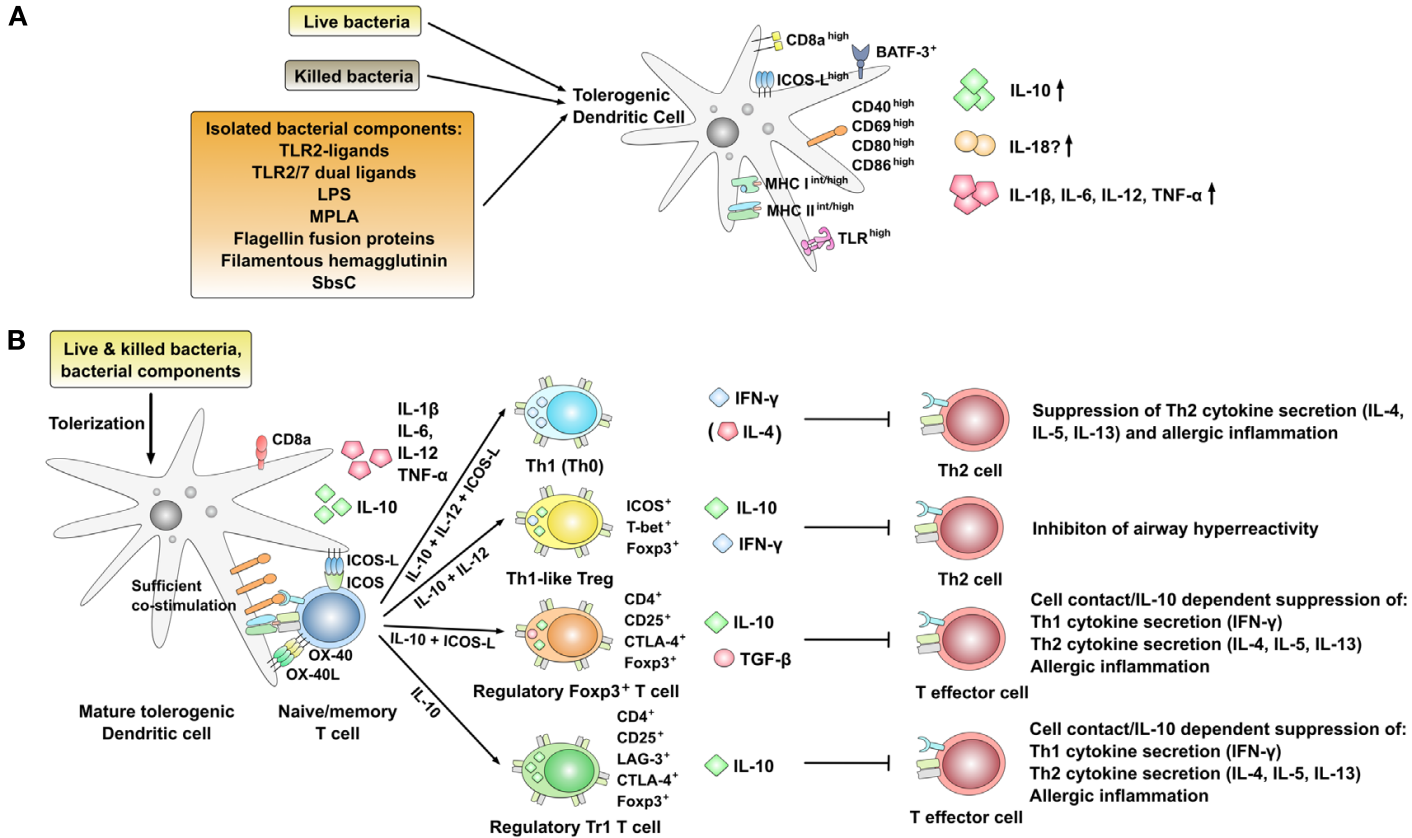
Phenotype and immune modulatory effects of interleukin-10 (IL-10) producing, mature tolerogenic DCs co-producing pro-inflammatory cytokines. **(A)** Strategies using live or killed bacteria and bacterial components resulting in pro-tolerogenic dendritic cell (DC) phenotypes characterized by the expression of high levels of co-stimulatory molecules as well as the co-production of IL-10 and pro-inflammatory cytokines [IL-1β, IL-6, IL-12, tumor necrosis factor alpha (TNF-α)]. Expression levels are indicated as follows: ^low^: low expression, ^int^: intermediate expression, ^high^: high expression, ^+^: positive for the indicated molecule; arrow up: increased production, arrow down: decreased production. **(B)** Regulation of T cell responses by DCs stimulated with bacteria or bacterial components inducing an IL-10-positive DC phenotype that is characterized by the co-production of IL-12 and high expression levels of co-stimulatory molecules. In this context, both the induction of different regulatory T cell subsets and Th1-biased effector cells have been described. ^+^: positive for the indicated molecule.

We have seen that immature DCs can be used to as tolerogenic DCs to suppress antigen-specific T cell responses. A regulatory, IL-10-producing DC phenotype is of special interest for the treatment of allergic diseases where the treatment aim is to downregulate allergen-specific Th2 responses and the associated clinical symptoms (allergen-specific Th2-responses, IgE-mediated mast cell and basophil activation, allergic inflammation). The remainder of this review will summarize the strategies used to generate such IL-10-producing DCs with a focus on IL-10 treatment, IL-10 transduction, and the usage of both whole bacteria and bacteria-derived components (see also Figures 2 and 3 and Table 1). Besides these strategies, several other factors have been described to promote the differentiation of IL-10-producing DC subsets in the context of allergies. Among others, these are endothelial splenic stroma (11), aryl hydrocarbon receptors (63), the phosphodiesterase-4 inhibitor roflumilast (75), and dexamethasone (16). Also induction of cyclic adenosine monophosphate was shown to result in upregulated expression of DC-derived IL-10, attenuated secretion of the pro-inflammatory cytokines TNF-α and IL-12, and impaired T cell stimulation by these DCs (76).

**Table 1:** Summary of the strategies reported in the literature to induce dendritic cell (DC)-derived interleukin-10 (IL-10) secretion.

Strategy		Reference	DC phenotype	DCs IL-10 positive?	Immunological effects of tolerogenic DCs	Suppression shown to be IL-10 dependent?
DC differentiation in the presence of IL-10		Koya et al. (1)	CD11c^low^ CD80^low^ CD86^low^ Reduced IL-12 production	Yes	Suppression of Th2 cytokines IL-4, IL-5, and IL-13 *in vitro* Decrease of airway hyperreactivity (AHR) and airway inflammation *in vivo*	Yes

		Li et al. (77)	CD86^low^ HLA-DR^low^ CD54^low^ CD40^int^ CD80^int^ Ig-like transcript-22/CD85j^high^ reduced levels of IL-6 and IL-12	Yes	Suppression of Th2-differentiation and Th2-cytokine secretion Activation of CD4^+^CD25^+^LAG-3^+^ CTLA-4^+^Foxp3^+^ IL-10-secreting Tregs Induction of allergen-specific tolerance	Yes and Treg cell contact dependent

		Bellinghausen et al. (78)	IL-10^+^ CTLA-4^+^ TGF-β^+^	Yes	IL-10- and TGF-β-dependent induction of regulatory T cells suppressing Th2 cytokine production	No, but programmed death-1 dependent

		Bellinghausen et al. (79)	CCL-18^+^	Yes	Suppression of IL-13, IL-5, and TNF receptor superfamily member 4 gene expression in CD4^+^ T cell:DC co-cultures	No, but CCL18 dependent

DC transduction with IL-10	Lentiviral transduction with CMV-promoter	Henry et al. (4)	CD40^int^ MHC II^int^ CD80^int^ CD86^int^ IL-12^low^	Yes	Prevention of eosinophilic airway inflammation, AHR, production of mucus, antigen-specific IgE and IgG1 antibody, and IL-4 production in a mouse model of experimental asthma	Yes and Treg dependent

	Plasmid vector	Nakagome et al. (80)	CD11c^+^ MHC II^low^	Yes	No induction of tolerogenic DCs or Treg, but overall suppression of function of CD11c antigen-presenting cells in the lung Prevention of eosinophilic airway inflammation *in vivo*	Not investigated

	Lentiviral transduction with DC-specific fascin promotor	Besche et al. (3)	MHC II^int^ CD86^int^ Unaltered IL-6 mRNA Lower IL-12p40 mRNA levels	Yes	Inhibition of ear swelling in mouse model of hapten-induced contact hypersensitivity	Not investigated

DC-derived exosomes		Kim et al. (81)	MHC II^high^ MHC I^int^ CD11c^int^ CD80^int^ CD86^int^	No	Suppression of delayed-type hypersensitivity responses and murine collagen-induced arthritis	No, but via MHC II-dependent pathway

		Ruffner et al. (82)	IA/IE^high^ H-2k^b int^ CD80^low^ CD86^int^ PD-L1^int^ PD-L2^low^ IL-12p70^−^ IL-23^−^ IL-6^+^	Not determined	Suppression of delayed-type hypersensitivity responses	CD80 and CD86 dependent

Bacteria	*Helicobacter pylori (live/extract)*	Engler et al. (83)	BATF3^+^ CD103^+^ CD11b^+^	Yes	Suppression of airway inflammation in a mouse model of allergic asthma	Yes, and IL18 basic leucine zipper ATF-like 3 (BATF3) dependent

	*Escherichia coli* 083	Súkeníková et al. (57)	CD83^high^ IDO^high^ TNF-α^+^ IL-6^+^	Yes	Increased expression of IL-10 and IL-17A in CD4 T cells	Not investigated

	*Bacillus* Calmette–Guérin	Bilenki et al. (84)	CD8a^high^ CD80^high^ CD86^high^ CD40^high^ IL-12^+^ TLR2^high^, TLR4^high^, TLR9^high^	Yes	Suppression of allergic airway eosinophilia, mucus overproduction, IgE production, and Th2 cytokine production *in vivo*	Yes, also IL-12 dependent

	*Listeria monocytogenes*	Stock et al. (85)	CD8a^+^ IL-12^+^	Yes	Induction of Th1-like ICOS^+^ Foxp3^+^T-bet^+^ Tregs co-producing both IL-10 and interferon gamma (IFN-γ)	Yes

	*Clamydia*	Han et al. (27)	CD8^high^ ICOS-L^high^ IL-10^high^ IL-12^high^	Yes	Inhibition of allergen-specific Th2 cell differentiation *in vitro* Inhibition of systemic and cutaneous eosinophilia *in vivo*	Yes, also IL-12 and ICOS-L dependent

Bacterial extracts	Heat killed *E. coli*	Pochard et al. (86)	IA^b high^ CD40^int^ CD80^high^ CD86^high^ IL-12^+^	Yes	Suppression of peanut-induced Th2 cytokine production and proliferation and induction of IFN-γ from mouse T cells	No, but myeloid differentiation primary response 88 (MyD88)/TIR-domain-containing adapter-inducing interferon-β, IL-12/IL23 p40, and IFN-γ dependent

Isolated bacterial components (TLR ligands)	Pam_3_CSK_4_	Tsai et al. (87)	Not investigated	Not investigated	Induction of CD8^+^CD25^+^ Foxp3^+^ Tregs that inhibited *Dermatophagoides pteronyssinus* 2-induced IL-4 production *in vitro*	Not investigated

	Dual TLR2/7-ligands	Laiño et al. (88)	IL-1β^low^ IL-6^+^	Yes	Suppression of Th2 cytokine secretion and DNP-induced, IgE- and Ag-specific mast cell degranulation *in vitro* Suppression of allergen-specific IgE production *in vivo*	Not investigated

	LPS	Ahrens et al. (89)	CD40^high^ CD80^high^ CD86^high^ IL-1β^+^ IL-12^+^ TNF-α^+^	Yes	Suppression of Th2 cytokine production and induction of Tr1-like cells *in vitro* No suppression of ovalbumin (OVA)-induced asthma *in vivo*	Yes

	LPS (plus IL-10 treatment)	Wakkach et al. (90)	CD11c^low^ B220^−^ CD45RB^+^	Yes	Increased eosinophilic airway inflammation and AHR, IL-5, and IL-13 secretion in bronchoalveolar lavage fluid in a mouse model of OVA-induced asthma	Not investigated

	Monophosphoryl lipid A	Schülke et al. (91)	CD40^+^ IL-1β^+^ IL-6^+^ TNF-α^+^	Yes	Boosting of OVA-specific IL-4 and IL-5 secretion, suppression of IFN-γ secretion in bone marrow-derived DC: DO11.10 CD4^+^ T cell co-cultures	Not investigated

	Flagellin fusion proteins	Schülke et al. (92–96)	CD40^+^ CD69^+^ CD80/86^+^ B7-H1^+^ B7-H4^+^ IL-6^high^	Yes	Suppression of Th1 and Th2 responses *in vitro* Suppression of sensitization and OVA-induced intestinal allergy *in vivo*	Yes

Isolated bacterial component (non TLR-ligands)	Flamentous hemagglutinin	McGuirk et al. (97)	MHC II^int^ CD40^int^ CD80^int^ CD86^int^ CCR5^int^ IL-12^low^ CCL3^low^	Yes	Suppression of Th1 cell proliferation and cytokine secretion, but not Th2-responses *via* differentiation of Tr1 Tregs	Yes

	SbsC:Bet v 1 fusion proten	Gerstmayr et al. (98)	CD40^int^ CD80^int^ CD86^int^ IL-12^+^	Yes	Induction of IL-10-producing CD25^+^Foxp3^+^CTLA-4^+^ Th0/regulatory T cells co-producing IFN-γ and IL-4	Not investigated, but IL-12 dependent

*Expression levels are indicated as follows: ^low^: low expression; ^int^: intermediate expression; ^high^: high expression; ^+^: positive for the investigated molecule but no expression level specified*.

### IL-10 Treatment

The simplest strategy to induce tolerogenic IL-10 DCs is to differentiate naive DCs in the presence of IL-10. Indeed, several studies reported IL-10-treated human or mouse DCs to induce antigen-specific anergy (99–101).

Immunologically, T cell tolerization (meaning the induction of antigen-specific CD4^+^CD25^+^ Treg cells) by IL-10-treated DCs requires a partially activated DC status commonly referred to as semimaturation (102, 103). In contrast to this, complete DC activation likely is more immunogenic than tolerogenic, resulting in the activation of effector T cell subsets (102, 103). This semi-mature status is characterized by high-expression levels of MHC, intermediate to low levels of co-stimulatory molecules, and a strongly reduced production of pro-inflammatory cytokines such as IL-12 (Figure 2A) (102). Moreover, the development of antigen-specific CD4^+^CD25^+^Foxp3^+^ Treg cells, inhibiting allergic responses, was shown to be dependent on the presence of both IL-10 and an inducible co-stimulator (ICOS)–inducible co-stimulator ligand (ICOS-L) interaction provided by DCs (Figure 2B) (32).

Here, the suppression of Th2-responses by the induced regulatory T cells was repeatedly described to occur *via* a cell contact-dependent and antigen non-specific manner (48, 104, 105). For example, DCs treated with IL-10 were shown to induce CD4^+^ T cells expressing the cytotoxic T lymphocyte-associated antigen 4 (CD152, CTLA4), capable of mediating tolerance in a cell contact-dependent manner (Figure 2B) (99, 106). In addition, some studies reported an increased expression of inhibitory molecules such as the Ig-like transcript-22/CD85 (77) on the surface of IL-10-treated DCs (Figure 2A).

The exact tolerogenic potential of IL-10-treated DCs seems to dependent on the experimental model used: while there are some reports suggesting that DCs treated with IL-10 may increase the secretion of Th2 cytokines (while suppressing Th1-responses) (107, 108), the majority of studies have described an efficient suppression of both mouse and human Th1- and Th2-responses by DCs treated with IL-10 (101).

The potency of IL-10-treated DCs to prevent the development of lung allergic responses in mice was demonstrated by Koya and colleagues (1). Koya et al. reported IL-10-treated DCs to suppress production of the Th2 cytokines IL-4, IL-5, and IL-13 *in vitro* and decrease both AHR and airway inflammation *in vivo* (1). Here, transfer of ovalbumin (OVA)-pulsed, IL-10-treated DCs into naive mice prevented the development of AHR, airway eosinophilia, reduced Th2 cytokine levels in bronchoalveolar lavage (BAL) fluid, and goblet cell metaplasia when challenged with the allergen (1).

In their hands, the IL-10-treated DCs displayed a tolerogenic phenotype, expressing lower levels of CD11c, CD80, and CD86, while producing lower amounts of IL-12 but significantly more IL-10 (Figure 2A) (1). Mechanistically, this endogenous IL-10 production of exogenously IL-10-treated DCs was shown to be required for their regulatory function since DCs from IL-10-deficient mice did not display regulatory function even when differentiated in the presence of IL-10 (1).

In accordance with these results, Li et al. did report IL-10-treated human DCs to induce tolerance in autologous T cells of patients with asthma (77). Phenotypically, the IL-10-treated DCs expressed reduced levels of the co-stimulatory and maturation markers CD86, human leukocyte antigen DR, and CD54, only modest reductions in CD40 and CD80, and reduced levels of the pro-inflammatory cytokines IL-6 and IL-12 (Figure 2A) (77). In contrast to this, expression levels of Ig-like transcript-22/CD85j, IFN-α1, IL-2, and IL-10 were strongly increased (Figure 2A) (77). In this context, the inhibitory receptor Ig-like transcript-22/CD85j was shown to have an important role both in the regulation of natural killer cells and T cells (109, 110) and the function of tolerogenic DCs (110).

In co-culture with autologous CD4^+^ T cells IL-10-treated DCs inhibited Th2 cell differentiation and production of Th2-related cytokines (IL-4, IL-5, and IL-13) otherwise driven by immunostimulatory DCs differentiated in the presence of TNF-α (77). Moreover, treatment of DCs with IL-10 led to a significant outgrowth and activation of CD4^+^CD25^+^LAG-3^+^CTLA-4^+^ Foxp3^+^ IL-10-secreting Tr1-type Tregs, and resulted in allergen-specific induction of tolerance in a contact-dependent manner which was critically dependent on expression of IL-10 by DC (Figure 2B) (77).

Although the tolerogenic capacity of IL-10-treated DCs is well described, Bellinghausen et al. reported treatment of DCs with IL-10 alone (in contrast to the efficient suppression of Th1 responses by IL-10-treated DCs) to be insufficient for the suppression of Th2-responses (78). In their hands, the induction of regulatory T cells with the ability to suppress Th2 cytokine production required at least two signals: IL-10 plus TGF-β (78). In their experimental system, the suppressive capacity of the IL-10 plus TGF-β-induced regulatory T cells was shown to be antigen-unspecific and strongly dependent on both cell–cell contact and the surface molecule programmed death-1 (PD-1) (78). Interestingly, neutralization of either IL-10, CTLA-4, or TGF-β had only marginal effects on the suppressive capacity of the induced CD4^+^CD25^+^ Tregs (78). Here, incubation of T cells with IL-10 alone instead of IL-10-treated DC did not lead to the generation of inducible Tregs (iTregs), suggesting that additional signals provided by the tolerizing DC are necessary for the generation of iTregs (78).

One such factor might be DC-derived CC-chemokine ligand 18 (CCL18). When performing a genome-wide analysis of gene expression in co-cultures of CD4^+^ T cells from patients with grass pollen allergy and either tolerogenic, IL-10-treated DCs or regular, mature allergen-pulsed DCs, Bellinghausen and coworkers could show that in DCs differentiated in the presence of IL-10 the only gene being upregulated was CCL18 (while many genes were downregulated) (79). These IL-10-treated, CCL18-producing DCs efficiently suppressed IL-13, IL-5, and TNF receptor superfamily member 4 (OX40) gene expression in CD4 T cell:DC co-cultures (79). Of note, exogenous addition of CCL18 to these co-cultures was sufficient to induce a similar inhibition of Th2 cytokine production compared to allergen-pulsed, IL-10-treated DCs (without affecting IFN-γ or IL-10 production) (79). In these co-cultures, neutralizing IL-10 did not reduce CCL18 production suggesting that factors other than IL-10 are involved in maintaining the enhanced CCL18 expression in IL-10-treated DCs (79). In a humanized mouse model of airway allergy, application of CCL18 inhibited airway reactivity and lung inflammation, preferentially attracting regulatory T cells over Th2 cells (79). Therefore, CCL18 was shown to be an important effector molecule of tolerogenic IL-10-treated DCs.

### IL-10 Transduction

Besides differentiating DCs in the presence of IL-10, several studies have described transduction of DCs with the IL-10 gene to result in DCs with tolerogenic properties. Here, IL-10-transduced DCs were shown to induce long-lasting, antigen-specific tolerance by induction of regulatory T cells (Figures 2A,B) (4).

Henry and coworkers reported a single intratracheal injection of OVA-pulsed, IL-10-transduced DCs to prevent eosinophilic airway inflammation, AHR, production of mucus, antigen-specific IgE and immunoglobulin G1 (IgG1) antibodies, and IL-4 as well as IFN-γ production in a mouse model of experimental asthma (Figure 2B) (4). These effects were shown to also depend on non-DC-derived IL-10 since IL-10-deficient mice treated with IL-10-transduced wild-type DCs were less well protected (4). Phenotypically, IL-10-transduced DCs displayed intermediate levels of cell surface maturation markers MHC II, CD40, CD80, and CD86 and secreted high amounts of IL-10, but no IL-12 (Figure 2A) (4). In contact with allergen-specific T cells, these semi-mature DCs induced both differentiation and proliferation of antigen-specific CD4^+^CD25^+^Foxp3^+^ IL-10-producing regulatory T cells in the mediastinal lymph nodes of animals treated with IL-10-transduced DCs (4). These effects were shown to be antigen-specific, since IL-10-transduced DCs, primed with the major house dust mite allergen *Dermatophagoides pteronyssinus* peptidase 1 (Der p 1), did not protect against OVA-induced airway allergy (4).

In line with these results, Nakagome et al. reported IL-10 gene delivery by plasmid transfer to suppress OVA-induced eosinophilic airway inflammation and AHR, suppressing the overall function of CD11c^+^ lung APCs in terms of antigen-presenting capacity, cytokine production, and transport of antigen to lymph nodes resulting in reduced Th2 responses (80).

In an attempt to optimize DC-derived IL-10 transduction, Besche et al. showed that the usage of the DC-specific fascin promoter for IL-10 overexpression in bone marrow-derived DCs (BM-DCs) to result in the generation of IL-10^+^IL-6^+^ DCs with reduced IL-12p40 mRNA expression (3). *In vivo* application of these IL-10-transduced BM-DCs efficiently inhibited ear swelling responses in a mouse model of hapten-induced contact hypersensitivity (3).

### Exosomes from IL-10-Treated DCs

Besides secreting immune modulatory cytokines, IL-10-treated DCs were also shown to secret exosomes with immune modulating capacity involved in the suppression of inflammatory and autoimmune responses (Figure 2A) (81).

Kim et al. reported exosomes isolated from either BM-DCs transduced *ex vivo* with an adenovirus expressing the IL-10 gene or BM-DCs treated with recombinant murine IL-10 protein to express high levels of MHC II, moderate levels of MHC I, CD11c, CD80, and CD86 on their surface (Figure 2A) (81). Upon periarticular administration, these exosomes were shown to suppress delayed-type hypersensitivity responses within both injected and untreated contralateral joints, while systemic injection suppressed the onset of murine collagen-induced arthritis and reduced the severity of established arthritis in a mouse model (Figure 2B) (81). Here, administration of isolated exosomes had comparable effects to the application of IL-10 transduced DC (81). Mechanistically, the suppressive capacity of the exosomes was shown to depend on surface expression of MHC II (81). The authors speculated that these exosomes may be able to bind and possibly fuse with endogenous cells (macrophages, or APCs) to subsequently modulate their activity (81).

These results were confirmed by Ruffner and colleagues which demonstrated that IL-10 treatment generates both DCs with a pro-tolerogenic phenotype and a population of immunosuppressive exosomes (82). Treatment of DC with IL-10 significantly downregulated surface expression of MHC I, MHC II, CD80, CD86, and programmed death ligand 2 (PD-L2) (Figure 2A) (82). In addition to the modified co-stimulatory profile of IL-10-treated DCs, exosomes derived from these DCs were shown to also contain reduced surface levels of CD80, PD-L1, and PD-L2 (Figure 2A) (82). Here, the suppressive capacity of both IL-10-treated DCs and exosomes derived from these cells in a mouse model of delayed-type hypersensitivity was shown to depend on CD80 and CD86, but not PD-L1 and PD-L2 expression (82).

### Live Bacteria

Several studies have described the potential of different bacteria to induce DC-derived IL-10 production (27, 84). Among others, *E. coli* 083 (57), *Helicobacter pylori* (83), *Clamydia* (27), *Listeria monocytogenes* (85), *Mycobacterium vaccae* (111), and *Bacillus* Calmette–Guérin (BCG) (84) were reported to induce DC-derived IL-10 secretion with immune modulatory potential for the treatment of allergic diseases. Of note, in contrast to IL-10-treated DCs, many of the available studies suggest that stimulation with either bacteria (live or killed) or isolated bacterial components induces both anti-inflammatory IL-10- and pro-inflammatory, Th1-promoting IL-12 secretion often alongside an enhanced expression of co-stimulatory molecules on the stimulated DCs (Figure 3A) (27, 84, 86, 98, 112).

Here, IL-10 secretion induced by bacteria can either prevent excessive inflammatory responses or suppress immune responses otherwise directed against the bacterium (27). In theory, this IL-10 induction by bacteria may be used to modulate immune responses in the host to unrelated antigens such as allergens (27).

In line with this, epidemiological and experimental studies revealed a strong inverse relationship between chronic *H. pylori* infection (which induces IL-10 secretion from DCs) and the risk of developing allergic asthma, hay fever, or eczema (113–115). Here, Engler and colleagues further investigated the mechanism underlying the protective effects of *H. pylori* (83). They reported extracts of *H. pylori* to prevent allergen-induced airway hyperresponsiveness, bronchoalveolar eosinophilia, pulmonary inflammation, and Th2 cytokine production in a mouse asthma model (Figure 3B) (83). Mechanistically, this suppression of Th2-responses was shown to require a heat-sensitive *H. pylori* component (possibly the *H. pylori* persistence determinants γ-glutamyl-transpeptidase GGT and the vacuolating cytotoxin VacA) and the production of IL-10 by basic leucine zipper ATF-like 3 (BATF3)-dependent CD103 and CD11b positive DCs infiltrating the lungs of protected animals (Figure 3A) (83). Moreover, both IL-18 and BATF3 were critically required for *H. pylori*-mediated protection against allergic responses (83). In contrast to this, suppression of Th2-responses was independent of regulatory T cells since antibody-mediated depletion of CD25^+^ Tregs had no effect on the suppression of Th2-responses (83). Interestingly, *in vitro* BM-DC-derived IL-10 secretion induced by *H. pylori* extracts was shown to depend on myeloid differentiation primary response 88 (MyD88) and toll-like receptor (TLR)2- but not TLR4-signaling (83).

Súkeníková et al. reported *E. coli* 083 to also induce increased gene expression and secretion of IL-10 in DC of newborns of healthy mothers in comparison to DCs derived from newbornes from allergic mothers (57). This higher IL-10 production was associated with lower levels of IL-4, IL-13, IFN-γ, IL-17A, and IL-22 in DC:CD4^+^ T cell co-cultures (Figure 3B) (57).

In line with these results, infection of mouse DCs with BCG resulted in a significant enhancement of both IL-10 and IL-12 production (84). Interestingly, BCG-stimulated DCs were characterized by a higher surface expression of CD8a, co-stimulatory molecules CD80, CD86, and CD40, and TLRs (Figure 3A) (84). Here, adoptive transfer of DCs from BCG-infected mice, but not DCs from naive mice, significantly inhibited established allergic airway eosinophilia, mucus overproduction, IgE production, and Th2 cytokine production (Figure 3B) (84). These protective effects of BCG-infected DCs were reversed by the application of either IL-10- or IL-12-neutralizing antibodies, showing both cytokines to be involved in the suppression of the allergic response (84).

Stock et al. described *L. monocytogenes* to induce CD8a^+^ DCs co-producing both IL-10 and IL-12 (Figure 3A) (85). These DCs mediated the differentiation of ICOS^+^Foxp3^+^ T-box transcription factor TBX21 (T-bet)^+^ Th1-like CD4^+^CD25^+^ Treg cells that themselves produced both IL-10 and the Th1 cytokine IFN-γ (Figure 3B) (85). Therefore, these cells combined features of both regulatory T cells and Th1 cells (85).

Han et al. reported the adoptive transfer of CD8^+^ICOS ligand (ICOS-L)^+^IL-10^+^IL-12^+^ DCs isolated from *Chlamydia*-infected mice (Figure 3A), but not those from naive mice, to inhibit OVA-induced systemic and cutaneous eosinophilia after intranasal challenge with OVA (27). *In vitro* DCs from *Chlamydia*-infected mice were shown to inhibit allergen-specific Th2 cell differentiation while promoting Th1 responses in an IL-10-, IL-12-, and ICOS-L-dependent way (Figure 3B) (27).

Taken together, these results show that in addition to inducing Th1-priming DCs, infection with different bacteria can result in the differentiation of tolerogenic, IL-10-producing DC subsets characterized by both high expression levels of co-stimulatory molecules and the co-production of pro-inflammatory cytokines (Figure 3A). Mechanistically, these more activated tolerogenic DCs were shown to suppress allergen-specific Th2-responses via the induction of either Th1, Th1-like Tregs, CD4^+^CD25^+^Foxp3^+^ Treg, or Tr1 cells (Figure 3B).

Aside from results obtained with live bacteria, also bacterial extracts and heat killed bacteria were described to suppress allergen-specific Th2-responses. Here, both IL-10-dependent and -independent mechanisms of immune modulation were described.

For example, Pochard et al. reported the addition of heat killed *E. coli* to peanut-pulsed DCs to suppress both peanut-induced secretion of the Th2 cytokines IL-4, IL-5, IL-13, and T cell proliferation while increasing IFN-γ production in a MyD88/TIR-domain-containing adapter-inducing interferon-β-dependent manner (86). Although stimulation of the DCs with heat killed *E. coli* did trigger DC-derived, TLR4-dependent IL-10 secretion, these effects of were not influenced by neutralization of IL-10 but shown to be dependent of IL-12/IL23 p40 and IFN-γ secretion (86). Therefore, the suppression of Th2-responses described by Pochard et al. is mediated by a TLR-mediated enhancement of Th1-responses, which in turn downregulate Th2-responses *via* IL-12 production (86).

### Bacterial Components

Besides whole bacteria and bacterial extracts, which are complex mixtures of different, potentially immune modulating components, some single bacterial components have been investigated for their DC tolerizing potential in the context of allergies (Figure 3A). For differentiation purposes, these components will be divided into TLR-ligands and non-TLR-ligands.

#### TLR Ligands

Bacteria- and virus-derived pathogen-associated molecular patterns are sensed by pathogen-recognition receptors and induce innate and subsequent adaptive immune responses. Due to their intrinsic capacity to activate innate immune cells, TLR ligands are interesting immune modulating components for the treatment of allergic diseases. Here, TLR2-, TLR3-, TLR4-, and TLR5-ligands have been described to induce tolerogenic DC subsets.

Bacterial lipopeptides such as the TLR2/6-ligand Pam_2_CysK_4_ have repeatedly been shown to induce tolerogenic DC and regulatory T cell responses (Figures 3A,B) (87, 116, 117). In addition, TLR2-ligands have the potential to induce a Th1-promoting cytokine milieu, enhance Ag presentation of endogenous peptides by DCs (117), and suppress IL-5, IL-13, and IFN-γ responses from human house dust mite-allergic patients (Figure 3B) (118).

Here, chemical conjugation of the TLR2-ligand Pam_3_CysK_4_ to OVA-derived CD8^+^ T cell peptide sequences resulted in a rapid and enhanced uptake in DCs (119). Moreover, dual TLR2/7-ligands combining the TLR2-ligand Pam_2_CysK_4_ and the synthetic TLR7-ligand CL264 into a single molecule were shown to induce strongly activated mDCs co-producing IL-10 and pro-inflammatory IL-6 (Figure 3A) (88). *In vitro*, these mDCs suppressed both DNP-induced, IgE- and Ag-dependent mast cell degranulation and IL-5 secretion from OVA-specific DO11.10 CD4^+^ TC (Figure 3B) (88). *In vivo* application of one of these ligands, CL531, was found to suppress allergen-specific IgE production in a mouse model of OVA-induced intestinal allergy, suggesting that such TLR2/7-ligands have the potential to induce Th1-biased immune modulation *in vivo* (88).

Ahrens et al. described LPS stimulation to strongly enhance IL-10 production from mouse BM-DCs (Figure 3A). In co-culture with allergen-specific naive CD4^+^ T cells, LPS-stimulated BM-DCs suppressed the secretion of Th1 and Th2 cytokines in an IL-10-dependent manner (89). Here, LPS priming of BM-DCs resulted in the differentiation of a Tr1-like T-cell subset upon co-culture of the primed DCs with naive T cells (89). Accordingly, Wakkach et al. reported LPS-, but not CpG-stimulation, to induce enhanced IL-10 secretion from IL-10-treated, CD11c^low^B220^−^CD45RB^+^ DCs (Figure 3A) (90). However, the suppressive capacity of LPS-primed BM-DC *in vitro* did not translate into suppression of allergic airway disease *in vivo* since intranasal administration of these LPS + IL-10-primed BM-DCs into mice was unable to prevent allergic airway inflammation in a mouse model of OVA-induced asthma (89). Here, vaccination with theseBM-DCs led to an even stronger eosinophilic airway inflammation and AHR accompanied by significantly increased levels of IL-5 and IL-13 in BAL fluid (89).

While LPS cannot be applied in humans due to its high toxicity and pyrogenicity, monophosphoryl lipid A (MPLA), a detoxified TLR4-ligand derived from *Salmonella minnesota*, is already applied as adjuvant in several vaccine formulations. Here, vaccines containing MPLA have been licensed or are in phase III trials including Fendrix (hepatitis B), Cervarix (human papillomavirus-16 and human papillomavirus-18), and RTS,S (malaria) (120–122). For the treatment of allergies, MPLA mixed with grass pollen extract was shown to result in enhanced production of IFN-γ and reduce the production of IL-5 in PBMC from grass pollen-allergic patients (123). In line with these results, MPLA was shown to induce mouse BM-DC activation (CD40 upregulation) and secretion of both pro- (IL-1β, IL-6, TNF-α) and anti-inflammatory (IL-10) cytokines *in vitro* (Figure 3A) (91). Of note, when MPLA-stimulated mouse BM-DCs were co-cultured with DO11.10 CD4^+^ T cells *in vitro* MPLA was shown to boost OVA-specific IL-4 and IL-5 secretion while dose-dependently suppressing IFN-γ secretion displaying a discrepancy between the results obtained *in vitro* and in clinical trials (91).

In addition, synthetic oligodeoxynucleotides containing CpG motifs (TLR9-ligands) either alone (124–126) or chemically linked to allergens (127–129) have been described to promote Th1 cytokine responses and decrease synthesis of IgE antibodies in allergic individuals. While these constructs were shown to induce the production of IL-12 and IL-18 from human moDCs (125) and induce IL-12, IFN-α, IFN-γ, IL-6, and IL-10 secretion from PBMCs (126), up to now no DC-derived IL-10 secretion has been reported upon application of these CpG-based vaccines.

In our own studies, we evaluated the induction of IL-10-producing mDCs using fusion proteins consisting of the recombinant TLR5-ligand flagellin A (rFlaA) from *L. monocytogenes* and either OVA from hen’s egg as a model allergen (rFlaA:OVA) (92–94), the major mugwort allergen *Artemisia vulgaris* allergen 1 (Art v1) (rFlaA:Artv1) (95), or the major birch pollen allergen *Betula verrucosa* allergen 1 (Bet v 1) (rFlaA:Betv1) (96). Such fusion proteins efficiently target TLR5^+^ immune cells, e.g., DCs, macrophages, and epithelial cells which take up, process, and present the fused antigen in the context of the flagellin-mediated cell activation.

Mouse bone marrow-derived mDCs stimulated with such fusion proteins were strongly activated [upregulation of CD40, CD69, CD80, CD86, programmed cell death 1 ligand 2 (B7-DC, CD273), PD-L1], displayed highly increased levels of the target receptor TLR5 on their cell surface, and secreted both pro- (IL-1β, IL-6) and anti-inflammatory (IL-10) cytokines (Figure 3A) (93, 95, 96). Interestingly, the non-fused mixture of both components (flagellin and allergen) did not have a comparable effect (93, 95, 96).

When co-cultured with allergen-specific CD4^+^ T cells, these DCs efficiently suppressed both allergen-induced Th1 and Th2 cytokine secretion *in vitro* (Figure 3B) (93, 95, 96). In this experimental setting, the flagellin fusion protein-mediated suppression of both Th1- and Th2-cytokine secretion was dependent on IL-10, since this effect was reversed when using either IL-10-neutralizing antibodies or IL-10-deficient mDC as APCs for the co-cultures (93).

*In vivo* vaccination with the rFlaA:OVA fusion protein efficiently protected mice from OVA-induced gastrointestinal allergy. Here, vaccination with rFlaA:OVA either intraperitoneal (93) or intranasal (92) was sufficient to prevent intestinal allergy induced by continuous challenge with OVA-containing food pellets. Interestingly, core body temperature, body weight, food up-take, and symptom scores were significantly improved in rFlaA:OVA-treated mice in comparison to the respective control groups (92, 93). This protective effect was associated with a reduction of Th2 cytokines in intestinal homogenates, suppression of systemic T cell immune responses, suppression of OVA-specific IgE-, and induction of OVA-specific IgG2a-responses (93). Vaccination with rFlaA and OVA alone or provided as a mixture did neither prevent allergic sensitization nor improve allergy symptom scores.

Mechanistically, stimulation of mDCs with flagellin fusion proteins was shown to result in a stronger uptake into mDCs (92, 96) accompanied by an increased resistance to microsomal digestion (92, 96). Interestingly, stimulation with rFlaA:Betv1 was shown to result in an increased metabolic activity of the stimulated mDCs characterized by a high rate of glycolysis followed by lactic acid fermentation, known as the Warburg effect (130). Further analysis suggested an activation of the mammalian target of rapamycin 1 complex in mDCs stimulated with the fusion protein (96). In this context, recent studies suggest that mTOR is not only a master regulator of cell metabolic function but also regulates innate immune responses (131).

Inhibition of the mTOR complex by pre-treatment of the cells with rapamycin dose-dependently suppressed the induction of anti-inflammatory IL-10 secretion by rFlaA:Betv1, but not pro-inflammatory cytokine secretion (IL-1β and IL-6). These findings show that interestingly, the immune-modulatory cytokine secretion, and therefore the DC tolerizing capacity, of this vaccine candidate was linked to the activation of mDC metabolism. Similar results were obtained for a fusion construct consisting of FlaA and the major mugwort allergen Art v 1 (95).

#### Non-TLR Ligands with DC-Tolerizing Potential

In the context of allergy treatment, some non-TLR ligands have been reported to induce DCs with tolerizing potential. Here, *Bordetella pertussis*-derived filamentous hemagglutinin was shown to induce the production of IL-10 by DCs promoting the differentiation of CD4^+^CD25^+^CCR5^high^CD28^low^CTLA-4^low^ IL-10- and IL-5-co-producing Tr1 cells (97).

Moreover, Gerstmayr and colleagues generated a recombinant fusion protein of a bacterial surface (S-layer) protein of *Geobacillus stearothermophilus* (SbsC) and the major birch pollen allergen Bet v 1 as a vaccine candidate to improve the treatment of birch pollen allergy. The SbsC:Bet v 1 fusion protein displayed reduced mediator-releasing capacity, while both preserving Bet v 1-T cell epitopes, and the potency to induce IFN-γ and IL-10 production in Bet v 1-specific Th2-biased T cell clones (98). DCs stimulated with the fusion protein were shown to have a semi-mature phenotype characterized by enhanced expression of CD40, CD80, and CD86 which were still lower than the levels induced by stimulation with LPS (Figure 3A) (98). Moreover, the SbsC:Bet v 1 fusion protein strongly increased DC-derived IL-10 and IL-12 secretion (98). Functionally, DCs matured with SbsC:Bet v 1 induced the IL-12- and IFN-γ-dependent differentiation of naive T cells into IFN-γ-producing T cells co-producing IL-4, suggesting a Th0 phenotype (Figure 3B) (98). In addition, naive T cells also differentiated into IL-10-producing CD4^+^CD25^+^Foxp3^+^CLTA-4^+^ regulatory T cells capable of active suppression, thus promoting the simultaneous differentiation of Th0/Th1 cells and regulatory T cells (Figure 3B) (98).

## Other Factors Contributing to the Inhibitory Capacity of DCs

Although many studies described the suppressive capacity of IL-10 secreting DCs, several other factors were reported to also mediate suppression of T cell responses without inducing DC-derived IL-10 production. Here, LPS- or polyriboinosinic-polyribocytidilic acid-induced production of IL-1β, indoleamine 2,3-dioxygenase (132), transforming growth factor-beta 1, vitamin D3 (133), corticosteroids, cyclosporine (134), as well as neuropeptides have been used to generate tolerogenic DCs (135, 136). Therefore, although the induction of IL-10 reproducibly leads to a tolerogenic phenotype of the induced DCs, other agents may also be used to generate DCs able to suppress T cells responses.

## Summary

Because of their potent T cell stimulatory as well as regulatory properties DCs have become a highly attractive tool in vaccine development to modulate antigen-specific immune responses.

While for cancer treatment and vaccination against infectious diseases the efficient induction of adaptive immune responses against the target antigens is the desired outcome when applying DC-based vaccination approaches, the therapy of autoimmune diseases, transplantat rejection, allergic reactions, or the control of chronic inflammation aims to induce DCs with tolerogenic properties.

Comparing IL-10-producing DCs induced either by IL-10-treatment or IL-10-transduction to IL-10-secreting DCs generated by stimulation with bacteria and bacterial components has revealed striking differences in the phenotype of the induced DCs and therefore the mechanism of tolerization: tolerogenic DCs generated by non-bacterial stimuli are arrested in an immature or semi-mature state, characterized by the production of reduced amounts of cytokines that promote T cell activation such as IL-12 and IL-6, a reduced capacity to present exogenous antigens, and the expression of lower amounts of co-stimulatory molecules.

In contrast to this, treatment of DCs with live or killed bacteria as well as isolated bacterial components results in the induction of both anti-inflammatory IL-10- as well as pro-inflammatory, Th1-promoting, IL-12 secretion often paralleled by an enhanced expression of co-stimulatory molecules on the stimulated DCs. This induction of Th1-priming, tolerogenic DCs generated by strongly activating stimuli was shown to suppress allergen-specific Th2-responses *via* the induction of either Th1-like Tregs, CD4^+^CD25^+^Foxp3^+^ Tregs, or Tr1 cells.

Therefore, while displaying the ability to directly suppress both Th1- and Th2-responses by different mechanisms, IL-10-producing DCs can efficiently modulate antigen-specific-specific immune responses *via* the induction of T cell subsets with regulatory functions. This makes IL-10-producing DCs promising therapeutics to improve the treatment of allergic diseases.

Over the past few years, we have started to understand the complex molecular mechanisms underlying the immune modulatory capacity of IL-10-producing DCs, identifying novel DC-derived factors that drive T cell tolerization such as DC-derived exosomes, CCL18, and inhibitory molecules like CTLA-4, OX40, Ig-like transcript-22/CD85, or PD-1.

Future studies will undoubtedly further increase our knowledge about the underlying immunological mechanisms allowing us to both refine and improve the application of DC-based vaccination approaches.

## Author Contributions

SS performed all research, prepared all the figures and tables, and wrote the manuscript.

## Conflict of Interest Statement

The author declares that the research was conducted in the absence of any commercial or financial relationships that could be construed as a potential conflict of interest.

## Abbreviations

AIT, allergen-specific immunotherapy; AHR, airway hyperreactivity; APC, antigen-presenting cell; AR, allergic rhinitis; Art v1, major mugwort allergen *Artemisia vulgaris* allergen 1; B7-DC, programmed cell death 1 ligand 2 (CD273); BAL, bronchoalveolar lavage; BATF3, basic leucine zipper ATF-like 3; BCG, *Bacillus* Calmette–Guérin*;* Bet v 1, major birch pollen *Betula verrucosa* allergen 1; BM-DCs, bone marrow-derived DCs; cAMP, cyclic adenosine monophosphate; CCL-3/18, CC-chemokine ligand 3/18; CCR5/7, CC chemokine receptor 5/7; CD, cluster of differentiation; cDC, conventional DC; CpG-ODN, oligodeoxynucleotides containing CpG motifs; CTLA-4, cytotoxic T lymphocyte-associated antigen 4 (CD152); DC, dendritic cell; Der p 1, major house dust mite allergen *Dermatophagoides pteronyssinus* peptidase 1; DTH, delayed-type hypersensitivity; FcεRI, high affinity IgE receptor Fc epsilon receptor I; Foxp3, forkhead box protein 3; HLA-DR, human leukocyte antigen DR; ICAM-1, intercellular adhesion molecule 1; ICOS, inducible co-stimulator; ICOS-L, inducible co-stimulator ligand; IDO, indoleamine 2,3-dioxygenase; IFN-α1, interferon alpha 1; IFN-γ, interferon gamma; Ig E/G, immunoglobulin E/G; IL, interleukin; IL-1RN, interleukin 1 receptor antagonist; IL-10R, interleukin 10 receptor; iTregs, inducible Tregs; Jak1, Janus kinase 1; LAG-3, lymphocyte-activation gene 3; LZT, low zone tolerance; MAP kinase, mitogen-activated protein kinase; mDC, myeloid DCs; MHC I/II, major histocompatibility complex I/II; MoDC, monocyte-derived DCs; MPLA, monophosphoryl lipid A; mTOR1, mammalian target of rapamycin 1; MyD88, myeloid differentiation primary response 88; OIT, oral immunotherapy; OX40, TNF receptor superfamily member 4 (CD134); OVA, ovalbumin; PAMP, pathogen-associated molecular pattern; PBMC, peripheral blood mononuclear cell; pDC, plasmacytoid DC; PD-1/2, programmed death-1/2; PD-L1/2, programmed death ligand ½; PDE4, phosphodiesterase-4; PRR, pathogen-recognition receptor; rFlaA, recombinant TLR5-ligand flagellin A; rFlaA:Artv1, recombinant fusion protein consisting of flagellin A and Art v 1; rFlaA:Betv1, recombinant fusion protein consisting of flagellin A and Bet v 1; rFlaA:OVA, recombinant fusion protein consisting of flagellin A and OVA; SbsC, surface (S-layer) protein of *Geobacillus stearothermophilus;* SbsC:Bet v 1, recombinant fusion protein consisting of SbsC and Bet v 1; SLIT, sublingual immunotherapy; SOCS-3, suppressor of cytokine signaling 3; STAT 1/5/6, signal transducer and activator of transcription 1/5/6; T-bet, T-box transcription factor TBX21; TGF-β, transforming growth factor beta; Th0/1/2/17, T-helper 0/1/2/17 cell; TLR, toll-like receptor; TRIF, TIR-domain-containing adapter-inducing interferon-β; TNF-α, tumor necrosis factor alpha; Tr1, regulatory T cell type I; Treg, regualory T cell; Tyk2, tyrosine kinase 2.
